# Supplementary material for: Circumcising daughters in Nigeria: To what extent does education influence mothers’ FGM/C continuation attitudes?
Source: PLOS Glob Public Health. 2022 Nov 18;2(11):e0000660. doi: 10.1371/journal.pgph.0000660 (PMC10021453; doi:10.1371/journal.pgph.0000660)
Supplement: S1 Text — (DOCX) [file pgph.0000660.s001.docx]

**SUPPLEMENTARY INFORMATION**

**Table A: Variables for the study and their codes**

| **Variables** | **Original coding in IPUMS - DHS** | **Recoding** | **Final codes** |
| --- | --- | --- | --- |
| FGM should continue | 1 = Continue  2 = Discontinue  3 = Depends/Doctor recommends |  | 1 = Continue  2 = Discontinue  3 = Depends/Doctor recommends |
|  |  |  |  |
|  |  |  |  |
|  |  |  |  |
| Mother’s education |  |  |  |
|  | 0 = No education  1 = Primary  2 = Secondary  3 = Higher |  | 0 = No education  1 = Primary  2 = Secondary  3 = Higher |
|  |  |  |  |
|  |  |  |  |
|  |  |  |  |
| FGM is an important religious tradition |  |  |  |
|  | 0 = Disagree  1 = Agree | 1 = 1  Else = 2 | 1 = Agree  2 = Disagree |
|  |  |  |  |
| Mother circumcised |  |  |  |
|  | 0 = No  1 = Yes |  | 0 = No  1 = Yes |
|  |  |  |  |
|  |  |  |  |
| Mother’s age | 20 = 15 – 19  30 = 20 – 24  40 = 25 – 29  50 = 30 – 34  60 = 35 – 39  70 = 40 – 44  80 = 45 - 49 | 20 – 30 = 1  40 = 2  50 = 3  60 = 4  70 = 5 | 1 = < 25  2 = 25-29  3 = 30-34  4 = 35-39  5 = 40+ |
|  |  |  |  |
|  |  |  |  |
|  |  |  |  |
|  |  |  |  |
|  |  |  |  |
| Religion |  |  |  |
|  | 2100 = Catholic  2900 = Other Christian, country-specific  1000 = Muslim  6100 = Traditional  9000 = Other | 2100&2900 = 1  1000 = 2  6100&9000 = 3  **3 was added to 2 due to small sample [32(0.6%)] | 1 = Christian  2 = Muslim |
|  |  |  |  |
|  |  |  |  |
|  |  |  |  |
|  |  |  |  |
| Marital Status |  |  |  |
|  | 10 = never married  21 = married  22 = Living together  31 = Widowed  32 = Divorced  33=Separated/not living together | 21 & 22 = 1  Else = 0 | 0 = Not in Union  1 = In Union |
|  |  |  |  |
|  |  |  |  |
|  |  |  |  |
| Place of Residence |  |  |  |
|  | 1 = Urban  2 = Rural | - | 1 = Urban  2 = Rural |
|  |  | - |  |
| Wealth Status |  |  |  |
|  | 1 = Poorest  2 = Poorer  3 = Middle  4 = Richer  5 = Richest | - | 1 = Poorest  2 = Poorer  3 = Middle  4 = Richer |
|  |  | - |  |
|  |  | - |  |
|  |  | - |  |
|  |  | - | 5 = Richest |


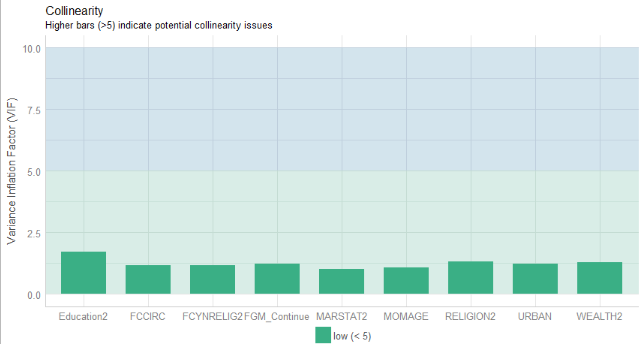


**Fig A: VIF showing multicollinearity among the independent variables.**

FCCIRC – Mother circumcised Education2 – Mother’s education

FCYNRELIG2 – FGM is an important religious practice FGM_Continue – FGM should continue

MOMAGE – mother’s age RELIGION2- Religion

URBAN – place of residence WEALTH2 – wealth quintile

MARSTAT2 – marital status

**Table B:** **Experience of circumcision by mother’s education among women who believe FGM should continue (n = 1495)**

|  | Ever circumcised | |  |
| --- | --- | --- | --- |
|  | **No** | **Yes** | **Total** |
|  | **%(n)** | **%(n)** | **%(n)** |
| Mother’s education |  |  |  |
| No education | 60.5 (608) | 39.5(397) | 100.0 (1005) |
| Primary | 43.9 (72) | 56.1(92) | 100.0 (164) |
| Secondary | 29.6(84) | 70.4(200) | 100.0 (284) |
| Higher | 11.9(5) | 88.1(37) | 100.0 (42) |
| Total (n) | 769 | 726 | 1495 |

% - row percentages

**Table C:** **Logistic regression results of circumcised daughters by background characteristics (with actual p-values)**

| Background characteristics | Model 0 | Model 1 | Model 2 | Model 3 | p- values for Model 3 |
| --- | --- | --- | --- | --- | --- |
|  | **OR[95%CI]** | **OR[95%CI]** | **OR[95%CI]** | **OR[95%CI]** |  |
|  |  |  |  |  |  |
| FGM/C should continue |  |  |  |  |  |
| Continue |  | 1.00 | 1.00 | 1.00 |  |
| Depends/Doctor recommends |  | 0.07[0.05,0.11]*** | 0.07[0.05,0.11]*** | 0.09[0.06,0.16]*** | **<0.001** |
| Discontinue |  | 0.02[0.016,0.029]*** | 0.03[0.02,0.04]*** | 0.05[0.04,0.08]*** | **<0.001** |
| Mother’s education |  |  |  |  |  |
| No education |  | 1.00 | 1.00 | 1.00 |  |
| Primary |  | 0.68[0.48,0.94]* | 0.83[0.48,1.46] | 0.95[0.52,1.73] | 0.869 |
| Secondary |  | 0.68[0.51,0.92]* | 1.04[0.64,1.69] | 1.28[0.74,2.21] | 0.376 |
| Higher |  | 0.43[0.26,0.70]*** | 1.59[0.53,4.76] | 1.83[0.58,5.79] | 0.307 |
| *FGM/C should continue:Education* |  |  |  |  |  |
| Continue:No education |  |  | 1.00 | 1.00 |  |
| Depends:Primary |  |  | 1.74[0.57,5.34] | 1.48[0.44,4.98] | 0.524 |
| Discontinue:Primary |  |  | 0.60[0.30,1.21] | 0.61[0.29,1.28] | 0.191 |
| Depends:Secondary |  |  | 0.95[0.35,2.58] | 0.99[0.34,2.95] | 0.991 |
| Discontinue:Secondary |  |  | 0.47[0.26,0.85]* | 0.66[0.36,1.21] | 0.175 |
| Depends: Higher |  |  | 0.69[0.09,5.15] | 0.98[0.12,8.10] | 0.986 |
| Discontinue:Higher |  |  | 0.15[0.04,0.50]** | 0.28[0.08,0.98]* | **0.047** |
| Control variables |  |  |  |  |  |
| FGM/C is an important religious practice |  |  |  |  |  |
| Agree |  |  |  | 1.00 |  |
| Disagree |  |  |  | 0.69[0.50,0.93]* | **0.017** |
| Mother circumcised |  |  |  |  |  |
| No |  |  |  | 1.00 |  |
| Yes |  |  |  | 13.06[9.70,17.58]*** | **<0.001** |
| Mother’s age |  |  |  |  |  |
| < 25 |  |  |  | 1.00 |  |
| 25-29 |  |  |  | 0.65[0.48,0.88]** | **0.006** |
| 30-34 |  |  |  | 0.63[0.46,0.88]** | **0.006** |
| 35-39 |  |  |  | 0.72[0.50,1.03] | 0.074 |
| 40+ |  |  |  | 0.82[0.55,1.22] | 0.335 |
| Religion |  |  |  |  |  |
| Christian |  |  |  | 1.00 |  |
| Muslim^a^ |  |  |  | 4.54[3.03,6.79]*** | **<0.001** |
| Marital status |  |  |  |  |  |
| Not in Union |  |  |  | 1.00 |  |
| In Union |  |  |  | 2.50[1.31,4.76]** | **0.005** |
| Place of Residence |  |  |  |  |  |
| Urban |  |  |  | 1.00 |  |
| Rural |  |  |  | 1.79[1.21,2.65]** | **0.004** |
| Wealth Status |  |  |  |  |  |
| Poorest |  |  |  | 1.00 |  |
| Poorer |  |  |  | 0.71[0.51,1.00]* | **0.049** |
| Middle |  |  |  | 0.72[0.49,1.06] | 0.093 |
| Richer |  |  |  | 0.58[0.38,0.88]* | **0.011** |
| Richest |  |  |  | 0.53[0.32,0.88]* | **0.015** |
| Random Effect |  |  |  |  |  |
| Cluster Variance [SE] | 7.34[0.08] | 3.604[0.06] | 3.76[0.06] | 3.37[0.05] |  |
| ∆% Cluster variance | - | -50.9 | 4.3 | -10.4 |  |
| Model statistics |  |  |  |  |  |
| Marginal R-squared (%) | 0.00 | 32.5 | 32.7 | 49.9 |  |
| AIC | 4794.8 | 3715.1 | 3710.0 | 3305.9 |  |
| ICC (%) | 69.1 | 52.3 | 53.3 | 50.6 |  |
