# Supplementary material for: Circumcising daughters in Nigeria: To what extent does education influence mothers’ FGM/C continuation attitudes?
Source: PLOS Glob Public Health. 2022 Nov 18;2(11):e0000660. doi: 10.1371/journal.pgph.0000660 (PMC10021453; doi:10.1371/journal.pgph.0000660)
Supplement: Attachment — Submitted filename: Response to Reviewers.docx [file pgph.0000660.s003.docx]

**Authors’ Response**

**Journal Requirements**

1. Please include a complete copy of PLOS’ questionnaire on inclusivity in global research in your revised manuscript.

**Authors’ response**: Thank you for drawing our attention to this. A complete inclusivity questionnaire has been uploaded as supporting information.

2. Please amend your Financial Disclosure statement. If you did not receive any funding for this study, please simply state: “The authors received no funding for this work.”

**Authors’ response:** The financial disclosure statement has been updated.

3. Please update your competing interests statement. If you have no competing interests, declare, please state: “The authors have declared no competing interests exist.”

**Authors’ response:** The competing interest statement has been updated.

4. Please note that your Data Availability statement is currently missing a direct link to access each database.

**Authors’ response:** The link to access the data has been provided.

**Reviewer 1**

1. This is a well written manuscript. The background should be reduced to about 2 pages. Summarize paragraphs 1 and 2 and present in a paragraph. Do the same in paragraphs 6 and 7. Doing this will out study rationale clearly.

**Authors’ Response:** We deeply appreciate the feedback on our work. Authors were able to summarise paragraphs 1 and 2 as well as 6 and 7 as suggested. The introduction was reduced to 2 and a half pages.

2. Summarize the statistical analysis section

**Authors’ response:** Authors have summarized the statistical analysis section (lines 185 to 209).

3. Little language editing is needed

**Author’s response:** Thank you for drawing our attention to this. Authors have checked and made all necessary edits.

**Reviewer 2**

1. Line 66 FGM/C has no health benefit. It is however a clear violation of girls and women’s human rights 67 as it inflicts irreversible and devastating complications such as sexual dysfunction, obstetric 68 complications and psychological trauma among survivors [5],

   Authors could reference specific studies done on psychological aspects of FGM/C in Nigeria and elsewhere. See:

Berg, R. C., Denison, E. M. L., & Fretheim, A. (2010). Psychological, social and sexual consequences of female genital mutilation/cutting (FGM/C): a systematic review of quantitative studies. Norwegian Knowledge Centre for the Health Services.

Omigbodun, O., Bella-Awusah, T., Groleau, D., Abdulmalik, J., Emma-Echiegu, N., Adedokun, B., & Omigbodun, A. (2020). Perceptions of the psychological experiences surrounding female genital mutilation/cutting (FGM/C) among the Izzi in Southeast Nigeria. Transcultural psychiatry, 57(1), 212-227.

Omigbodun, O., Bella-Awusah, T., Emma-Echiegu, N., Abdulmalik, J., Omigbodun, A., Doucet, M. H., & Groleau, D. (2021). Escaping Social Rejection, Gaining Total Capital: The Complex Psychological Experience of Female Genital Mutilation/Cutting (FGM/C) Among The Izzi in Southeast Nigeria. BMC Reproductive Health

Mulongo, P., Hollins Martin, C., & McAndrew, S. (2014). The psychological impact of female genital mutilation/cutting (FGM/C) on girls/women’s mental health: a narrative literature review. Journal of Reproductive and Infant Psychology, 32(5), 469-485.

**Authors’ Response:** Thank you recommending these sources. Authors have added some references to capture the psychological aspects (see line 66) .

2. Line 84 SDG target 5.3. What does SDG mean?

**Authors’ response:** Thanks for drawing our attention to this. SDG means sustainable development goals and authors have made the necessary updates in the text (see line 75)

3. Line 102 Social and behavioural change towards FGM/C has been a major tool to suppressing the practice. Need to be more specific here. What kind of change are you referring to?

**Authors’ response:** The change refers to both behavioural and attitudinal change regarding FGM/C. The paragraph has been updated to make things more clearer.

4. Line 125 A plausible pathway linking maternal education to daughter’s circumcision is through attitudes towards FGM/C. This statement needs to be substantiated in term of the rational as well as supporting references. What about daughter’s education?

**Authors’ response:** Thanks for drawing our attention to this. The preceding paragraph addresses the rationale for stating this linkage (lines 100-103). That maternal education influences women’s attitudes and their attitudes also affects their decision to circumcise their daughters. References have been inserted in lines 113-114 as well.

5. Line 131 The study produces useful insight on how education influence FGM/C which could 132 aid in designing public health educative programs and interventions targeted at addressing FGM/C 133 practices in Nigeria. This sentence goes in the conclusion section not the introduction.

**Authors’ response:** We have moved this to sentence to the conclusion section. See lines 413-415.

6. The interaction between low education level, positive attitude towards FGM and religion as predictors of FGM practice point to the need to discuss the implications of these variables for public health action; in particular, how girls and women can be educated on the deleterious effect of FGM even if they do not access higher level of education. How can religious leader, in particular Muslim leaders, play an education role to help end FGM? How can other types of education on FGM for mothers, fathers and daughter impact the attitude towards FGM?

**Authors’ response:** Thank you for this wonderful suggestion. We have incorporated this into our work. We find that the specifics to “how” Muslim leaders can play an educational role to help and how other types of education on FGM for mothers, fathers and daughters impact the attitude towards FGM require further research that falls beyond the scope of this study. We acknowledge the importance of the answers to these questions to addressing FGM.

One of my main problem is that the discussion section is underdeveloped when it comes to discussing the results in the light of the literature especially relating to the interaction of the roles played by education, attitudes (personal and cultural) and personal experience of the mother with own FGM. It seems a bit simplistic to focus the discussion only on anti-FGM policy. While policies and anti FGM laws are important they are not enough. Authors data suggest that attitudes, level of education and personal experience with FGM are key. Some qualitative studies have investigated these factors in different countries and should be discussed to enrich and substantiate the discussion section. For example, the mother’s personal experience with FGM was very important in determining attitude of some parents, who refuse to cut their daughter in a context of universal practice of FGM in Guinea. See:

Doucet, M. H., Delamou, A., Manet, H., & Groleau, D. (2020). Beyond will: the empowerment conditions needed to abandon female genital mutilation in Conakry (Guinea), a focused ethnography. BMC Reproductive health, 17(1), 1-15.

**Authors’ response:** Authors have updated the discussion section to capture issues the interactions identified in the study. See lines 331 – 404.

7. Authors should suggest the aspects of sociocultural dimension that would need to be studied in relation to these variables. For example, do future studies need to be comparing women with same level of education (high vs low), attitudes towards FGM (for vs against FGM vs ambivalent), self-experience of FGM (positive, negative, traumatic) to see what sociocultural process and context come into play to influence their decision not to cut their daughters. I this context looking at ‘positive deviants’ proved to be heuristic in understanding sociocultural process that come into play and could guide future strategies to eradicate FGM.

**Authors’ response:** Authors have incorporated this into the work. See lines 376-381.

8. Line 375. our findings deviate from the conclusions by De Cao and Giulia [23] who focused on the impact of the 376 Universal Primary Education programme on daughter’s circumcision and FGM/C attitudes.

   What do you mean here? Please clarify.

**Authors’ response:** Authors have clarified this in lines 360-367.

9. Also, what are the implications of these results for global public health?

**Authors’ response**: Authors have incorporated this in the concluding paragraph of the discussion (see lines 398-404).
