# Supplementary material for: Circumcising daughters in Nigeria: To what extent does education influence mothers’ FGM/C continuation attitudes?
Source: PLOS Glob Public Health. 2022 Nov 18;2(11):e0000660. doi: 10.1371/journal.pgph.0000660 (PMC10021453; doi:10.1371/journal.pgph.0000660)
Supplement: Attachment — Submitted filename: Response to Reviewers 2.docx [file pgph.0000660.s004.docx]

**AUTHORS’ RESPONSE TO COMMENTS**

**Article title:** Circumcising daughters in Nigeria: To what extent does education influence mothers' FGM/C continuation attitudes?

**Journal Requirements**

1. Please amend your online Financial Disclosure statement. If you did not receive any funding for this study, please simply state: “The authors received no specific funding for this work.”

**Authors’ response:** Authors have amended the online Financial Disclosure Statement

2. Please review your reference list to ensure that it is complete and correct. If you have cited papers that have been retracted, please include the rationale for doing so in the manuscript text, or remove these references and replace them with relevant current references. Any changes to the reference list should be mentioned in the rebuttal letter that accompanies your revised manuscript. If you need to cite a retracted article, indicate the article’s retracted status in the References list and also include a citation and full reference for the retraction notice.

**Authors’ response:** Authors have updated the reference list. We added the following references:

Okeke T, Anyaehie U, Ezenyeaku C. An overview of female genital mutilation in Nigeria. Ann Med Health Sci Res. 2012;2(1):70–3.

Muteshi JK, Miller S, Belizán JM. The ongoing violence against women: Female Genital Mutilation/Cutting. Reprod Health. 2016 Apr 18;13(1).

Kimani S, Muteshi-Strachan J, Njue C. Health impacts of female genital mutilation/cutting: A synthesis of the evidence. Reprod Health [Internet]. 2016 Jan 1 [cited 2022 Sep 11]; Available from: https://knowledgecommons.popcouncil.org/departments_sbsr-rh/637

Ogah J, Kolawole O, Awelimobor D. High risk human papilloma virus (HPV) common among a cohort of women with female genital mutilation. Afr Health Sci [Internet]. 2019 Dec 1 [cited 2022 Sep 11];19(4):2985. Available from: /pmc/articles/PMC7040333/

We excluded the following references:

Zurynski Y, Phu A, Sureshkumar P, Cherian S, Deverell M, Elliott EJ. Female genital mutilation in children presenting to Australian paediatricians. Arch Dis Child. 2017;102(6):509–15.

Okagbue HI, Ijezie OA, Samusenkov VO, Erondu EC, Eze GA. Female life expectancy, maternal mortality, fertility and birth rates of female genital mutilation high prevalence countries. Sci African. 2020;10(e00647):e00647.

**Reviewer comments**

**Reviewer 3**: The authors stated that the study considered “youngest daughters who were only born in the last five years preceding the 2018 DHS”. Although this may reflect the most recent prevalence of the practice in Nigeria, in other countries such as The Gambia, the recent DHS reported that while the younger population of children between 0 to 14 years reveals a lower number of 51 percent, 27 percent of children between the age of 0 and 4 years old have been subjected to FGM/C. So, the practice of FGM/C could even happen as soon as the child is born usually at home and not reported by parents for fear of the law, which may not be captured in the most recent DHS.

**Authors’ response**: Thank you and we agree with everything mentioned above. Yes, to ensure that information on attitudes towards FGM/C continuation among mothers during the time of the survey rightly aligns with the circumcision decision of their daughters, we decided to limit our sample to the most recent female birth – youngest daughters born in the last five years preceding the survey. An advantage for doing this was to reduce biases that could lead to reverse causality (lines 143-148). Meanwhile, it is true that some of these may not be reported and we have acknowledged this as a limitation (line 419-421).

**Reviewer 3: Line 304-305:** There were also higher odds of 2.50 of being circumcised if the mother was in union compared to those whose mothers were not in union. What could be the probable explanation (s) for this finding?

**Authors response**: Please find in lines 396 - 400, the explanation for the finding.
